# Community Asset Density and Past-Year Mental Health Symptoms Among Youths

**DOI:** 10.1001/jamanetworkopen.2024.34923

**Published:** 2024-09-20

**Authors:** Nicholas Szoko, Aniruddh Ajith, Kristen Kurland, Alison J. Culyba

**Affiliations:** 1 Division of Adolescent and Young Adult Medicine, University of Pittsburgh Medical Center Children’s Hospital of Pittsburgh, Pittsburgh, Pennsylvania; 2 Department of Pediatrics, University of Pittsburgh School of Medicine, Pittsburgh, Pennsylvania; 3 School of Architecture, Carnegie Mellon University, Pittsburgh, Pennsylvania; 4 H. John Heinz III College, Carnegie Mellon University, Pittsburgh, Pennsylvania

## Abstract

**Importance:**

Many youths experience mental health challenges. Identifying which neighborhood and community factors may influence mental health may guide health policy and practice.

**Objective:**

To explore associations between community assets (eg, schools, parks, libraries, and barbershops) and past-year mental health symptoms among youths.

**Design, Setting, and Participants:**

This cross-sectional study leveraged 3 datasets, which were linked by 26 zip codes: the Western Pennsylvania Regional Data Center, the Child Opportunity Index 2.0 database, and the Allegheny County Youth Risk Behavior Survey (YRBS). The YRBS was administered during the study period in 2018 to youths across 13 high schools in Allegheny County, Pennsylvania; the study dates were from October 15 to October 19, 2018. Dates of analysis were from August 1, 2023, to July 15, 2024.

**Exposures:**

Asset density in each zip code across 8 asset categories (transportation, education, parks and recreation, faith-based entities, health services, food resources, personal care services, and social infrastructure) was calculated.

**Main Outcomes and Measures:**

The main outcomes were mental health measures included in the past 12 months, which comprised feelings of hopelessness (feeing so sad or hopeless that you stopped doing activities), nonsuicidal self-injury (hurt yourself on purpose without wanting to die), and suicidal ideation (seriously considered attempting suicide). All were operationalized to any or none. Data were analyzed using multivariable generalized linear mixed models and were adjusted for age, sex assigned at birth, race and ethnicity, and identification as sexually or gender diverse.

**Results:**

Among 6306 students who were eligible for the YRBS based on their enrollment in participating high schools, 4487 students completed surveys, and 2162 were included in the analytic sample (mean [SD] age, 15.8 [1.2] years; 1245 [57.6%] were assigned female sex at birth). Over one-third of the participants (811 [37.5%]) reported past-year feelings of hopelessness; 587 (27.2%), past-year nonsuicidal self-injury; and 450 (20.8%), past-year suicidal ideation. High total asset population density (adjusted odds ratio [AOR], 0.85 [95% CI, 0.75-0.97]; *P* = .01), as well as population density of transportation assets (AOR, 0.77 [95% CI, 0.66-0.90]; *P* < .001), educational resources (AOR, 0.78 [95% CI, 0.67-0.92]; *P* = .002), and health services (AOR, 0.74 [95% CI, 0.60-0.91]; *P* = .006), were associated with lower odds of past-year hopelessness after adjusting for covariates. There were no correlations between asset density, Child Opportunity Index, and other mental health measures.

**Conclusions and Relevance:**

The findings of this cross-sectional study suggest that access to certain community assets was associated with lower odds of feelings of hopelessness among youths. Ongoing work is needed to characterize other forms of social and cultural capital, which may mitigate negative mental health outcomes among adolescent youths.

## Key Points

**Question:**

What types of community assets (eg, schools, parks, libraries, and barbershops) may be associated with youth mental health?

**Findings:**

In this cross-sectional study of 2162 adolescent youths surveyed across Allegheny County, Pennsylvania, living in zip codes with a high density of transportation assets, educational resources, and health services was associated with lower odds of past-year feelings of hopelessness.

**Meaning:**

These findings suggest that community assets offered important touchpoints for adolescents that may be leveraged to support youths’ mental health.

## Teaser

This cross-sectional study assessed which community asset categories may be associated with youth mental health symptoms.

## Introduction

Many youths in the US experience mental health challenges. In 2021, 42% of US high school students felt persistently sad or hopeless, and 22% had seriously considered suicide.^1^ Poor mental health in adolescence exerts wide-reaching impacts on overall health and well-being. Feelings of hopelessness may predict future thoughts of suicide and increase risk for violence perpetration and delinquency.^2^ Youths who participate in nonsuicidal self-injury (NSSI) are more likely to develop substance use disorders and report risky sexual behaviors.^3^ Risk and protective factors related to youth mental health have been described at individual, interpersonal, and community levels.^4,5,6^ There have thus been growing calls for cross-sector research, which considers adolescent well-being within a broader ecologic framework.^7,8^

Community assets, including parks, libraries, community centers, and salons or barbershops, represent important touchpoints for youths that may bolster mental health.^9,10,11^ Community gardens and green spaces have been associated with greater psychologic empowerment and reduced psychologic distress.^12,13,14,15,16^ Public libraries provide a safe and inclusive space for socialization and access to mental health resources, which may foster an increased sense of belonging.^17^ Barbershops and hair salons are cultural assets among Black communities and may act as nexuses for community-based mental health initiatives.^18,19^ Resources related to basic needs (eg, food security, stable housing) may also play a key role in supporting mental health service utilization.^20,21,22^

Geographic information systems involve mapping, visualizing, and analyzing spatial data patterns and have emerged as a powerful strategy in public health and medicine.^23,24^ Geographic information systems encompass numerous methods and leverage data from various sources. Geographic information system studies of green space may use satellite-derived variables such as a normalized difference vegetation index, green cover, and park density.^14^ Density-based measures have also been used to understand relationships between environmental features and health. For example, alcohol outlet density has shown associations with community violence,^25^ and concentration of fast food establishments has been associated with elevated body mass index.^26^ Given their potential for understanding the dynamic environments in which youths live and play, geospatial methods are particularly salient for understanding mental health access and outcomes.^27^

Composite indices, such as the Child Opportunity Index (COI)^28^ and the Social Deprivation Index,^29^ represent additional geospatial measures that have been used to explore ecologic influences on adolescent health. Derived primarily from census-level metrics (eg, percentage living in poverty, educational attainment), such indices allow for grading of zip codes or census tracts across specified parameters, generating a summary score for socioeconomic context.^30^ These measures have been associated with a variety of adolescent psychologic outcomes, including depressive symptoms, subjective well-being, and externalizing behaviors.^31,32^ Although these indices provide some insight into place-based determinants of mental health, they may inadvertently reinforce assumptions related to historic forms of oppression. Limitations in resolution (ie, the level of measurement) as well as the summarization strategy used (ie, ranking, normalization) may obscure variability within specific indicators or across smaller geographic areas.^30^ Many indices assess areas through a lens of deprivation, which fails to acknowledge the strength and vitality of communities.

Considering these challenges, we sought to leverage novel geographic information system methods to better understand associations between multiple types of community assets (eg, schools, parks, libraries, and barbershops) and youth mental health. Specifically, we examined associations between the density of community assets and youth hopelessness, NSSI, and suicidal ideation. Our primary goal was to better understand which community assets may serve as protective factors for youths’ mental health to inform future prevention and intervention programs.

## Methods

### Study Design and Data Sources

This cross-sectional study used data from the Western Pennsylvania Regional Data Center (WPRDC), the COI 2.0 database, and the Allegheny County Youth Risk Behavior Survey (YRBS) (eTable 1 in Supplement 1). Data from the WPRDC were accessed online as a comma-separated values text file with latitude and longitude data for over 30 000 assets in Allegheny County, Pennsylvania, compiled from January 1, 2019, to March 15, 2020, and active as of March 1, 2020, prior to the onset of the COVID-19 pandemic.^33^ The COI 2.0 database was downloaded directly from the host web page and included the population younger than 18 years in each zip code, derived from 2015 American Community Survey estimates and zip code COI scores across 3 domains: education, health and environment, and social and economic.^34^ The COI scores were aggregated by zip code boundaries from January 15, 2020, to align with asset data from the WPRDC. The Allegheny County YRBS was administered during the study period in 2018 using paper-and-pencil surveys to 9th through 12th grade students across 13 high schools in Pittsburgh, Pennsylvania, in partnership with the Allegheny County Health Department and Pittsburgh Public Schools; the study dates were from October 15 to October 19, 2018. The current study used data from respondents living in zip codes with at least 5 youths sampled. Comparisons between youths with and without missing zip code data are presented separately (eTable 2 in Supplement 1). Parents or guardians received informational letters prior to the YRBS survey with the option to opt out of their child’s participation by signing and returning the letter, and students could also decline to participate. All students who were present in school on the day of survey administration and whose parents or guardians had not opted out were eligible to participate. This study was deemed exempt from review by the University of Pittsburgh Institutional Review Board because it used deidentified data. This study was conducted in accordance with the Strengthening the Reporting of Observational Studies in Epidemiology (STROBE) reporting guideline.

### Measures

#### Demographics

Youths reported their age in years and the sex (female or male) that was listed on their birth certificate. Race was measured by asking youths to select all categories that applied, including American Indian or Alaska Native, Asian, Black or African American, Native Hawaiian or Other Pacific Islander, White, and other (for which no racial categories were specified). Ethnicity was assessed with a single item regarding Hispanic or Latino heritage (self-reported yes or no). A collapsed race and ethnicity variable was used in analyses, which included Hispanic, multiracial, or other; non-Hispanic Black; or non-Hispanic White. Youths who were sexually and gender diverse included those reporting any sexual identity other than heterosexual or any gender identity other than cisgender, using a 2-step measure of gender identity.^35,36^ Race and ethnicity and sexual and gender diversity were included in the study due to known inequities related to mental health and well-being.

#### Asset Density

Assets were classified into 8 categories that were hypothesized to be associated with mental health based on a review of existing literature^9,10,12,13,14,15,16,17,18,19,37,38^: transportation, education, parks and recreation, faith-based entities, health services, food resources, personal care services, and social infrastructure (eTable 3 in Supplement 1). There were 9338 (28.4%) of 32 938 assets that did not fall into 1 of these categories and were only included when calculating total asset density. Assets were visualized as point overlays on choropleth maps. In addition, assets were summarized by calculating the number of assets per zip code and dividing by the estimated population under 18 (density per population). We obtained asset density *z* scores by normalizing across the 26 zip codes included in the analysis.

#### COI

The COI included 3 domains, each made up of multiple indicators obtained from census-level data: education (eg, high school graduation rate), health and environment (eg, walkability), and social and economic (eg, employment rate). The COI scores were obtained by ordering more than 72 000 census tracts across indicators and assigning a score from 1 to 100 based on percentile rank, with higher scores indicating more favorable opportunity. The COI scores were normalized to generate *z* scores across zip codes included in the study sample.

#### Mental Health

Mental health measures included feelings of hopelessness, NSSI, and suicidal ideation in the past 12 months. Hopelessness was assessed with a single item that asked youths to indicate whether they experienced sadness or hopelessness that limited usual activities for 2 weeks or more (yes or no). Youths indicated NSSI by reporting the frequency with which they hurt themselves on purpose without wanting to die; responses were operationalized to a binary indicator variable (any or none). For suicidal ideation, youths were asked whether they had seriously considered attempting suicide (yes or no).

### Statistical Analysis

Dates of analysis were from August 1, 2023, to July 15, 2024. Geospatial analyses were performed in ArcGIS Pro, version 3.1 (Esri), and statistical analyses were completed in R, version 3.16.0 (R Project for Statistical Computing). Choropleth maps were created to display the percentage of youths reporting each mental health measure by zip code. To identify potential hotspots, we also conducted multivariate clustering analyses (eFigure 2 in Supplement 1). Quantitative geospatial data, including asset density and COI, were appended to the YRBS dataset according to respondent zip code. We used multivariable generalized linear mixed models to explore associations among asset density, COI, and each mental health measure, adjusting for age, sex assigned at birth, race and ethnicity, and identification as sexually or gender diverse. Models used a logit-link function and accounted for school-level clustering with a random intercept. Covariates were selected based on prior literature^1^ and hypothesized association with mental health measures. In asset density models, we included COI as a covariate to control for neighborhood contextual factors. We also examined correlations between total asset density and COI using Pearson product moment correlation. As an additional sensitivity analysis, we assessed associations between spatial asset density (calculated by dividing the number of assets by zip code area) and mental health measures given that certain asset categories (eg, parks and recreation) may depend on geographic area. We also conducted regression analyses using nationally normed COI *z* scores. Model fit was assessed by reviewing bayesian information criteria. Residual plots approximated a normal distribution without evidence of underdispersion or overdispersion. Variance inflation factors (all <3) did not suggest multicollinearity. Results of regression are presented as adjusted odds ratios (AORs) with 95% CIs. Unadjusted analyses and alternative multilevel models are available in eTables 4 and 5 in Supplement 1.

## Results

Out of 6306 students enrolled, 4487 completed surveys (71.2% response rate), with 280 surveys excluded due to being unreadable or too incomplete for analysis (eFigure 1 in Supplement 1). A total of 2162 youths across 26 zip codes were included in the analysis (Table 1). The mean (SD) age was 15.8 (1.2) years; 1245 (57.6%) were assigned female sex and 899 (41.6%) were assigned male sex at birth. Among the participants, 25 (1.2%) reported being American Indian or Alaska Native; 88 (4.1%), Asian; 610 (28.2%), Black or African American; 181 (8.4%), Hispanic or Latino; 9 (0.4%), Native Hawaiian or Other Pacific Islander; 1035 (47.9%), White; and 375 (17.3%), other racial identity. One-quarter of the participants (552 [25.5%]) identified as being sexually or gender diverse. Over one-third (811 [37.5%]) reported feelings of hopelessness in the past 12 months; NSSI and suicidal ideation were also common (NSSI: 587 participants [27.2%]; suicidal ideation: 450 participants [20.8%]).

**Table 1.** Demographic Characteristics of Patients

Characteristic	Values (N = 2162)^a^
Age, mean (SD), y	15.8 (1.2)
Sex assigned at birth	
Female	1245 (57.6)
Male	899 (41.6)
Race^b^	
American Indian or Alaska Native	25 (1.2)
Asian	88 (4.1)
Black or African American	610 (28.2)
Native Hawaiian or Other Pacific Islander	9 (0.4)
White	1035 (47.9)
Other^c^	375 (17.3)
Hispanic or Latino ethnicity^d^	181 (8.4)
Sexual or gender diversity^e^	552 (25.5)
Mental health measure	
Hopelessness	811 (37.5)
NSSI	587 (27.2)
Suicidal ideation	450 (20.8)

Abbreviation: NSSI, nonsuicidal self-injury.

^a^ Data are presented as No. (%) of patients unless indicated otherwise. Due to missing data, numbers or percentages may not match the total number of patients or sum to 100%.

^b^ Respondents were instructed to select all identities that applied.

^c^ Other did not include specific racial categories.

^d^ Assessed as a single item with a yes or no response.

^e^ Includes those reporting any sexual identity other than heterosexual or any gender identity other than cisgender.

Choropleth maps for mental health measures are shown in the Figure. Adjusting for covariates, high total asset density was associated with lower odds of hopelessness using both population and spatial density measures (population: AOR, 0.85 [95% CI, 0.75-0.97]; *P* = .01; spatial: AOR, 0.87 [95% CI, 0.78-0.98]; *P* = .02). Similar findings were observed for 3 asset density subcategories, including transportation (population: AOR, 0.77 [95% CI, 0.66-0.90]; *P* < .001; spatial: AOR, 0.83 [95% CI, 0.73-0.95]; *P* = .006), education (population: AOR, 0.78 [95% CI, 0.67-0.92]; *P* = .002; spatial: AOR, 0.87 [95% CI, 0.77-0.98]; *P* = .02), and health services (population: AOR, 0.74 [95% CI, 0.60-0.91]; *P* = .006; spatial: AOR, 0.80 [95% CI, 0.67-0.95]; *P* = .01) (Table 2). Faith-based asset population density (AOR, 0.85 [95% CI, 0.73-0.99]; *P* = .04) and food resource spatial density (AOR, 0.89 [95% CI, 0.81-0.98]; *P* = .02) were also associated with lower odds of hopelessness.

**Figure.**
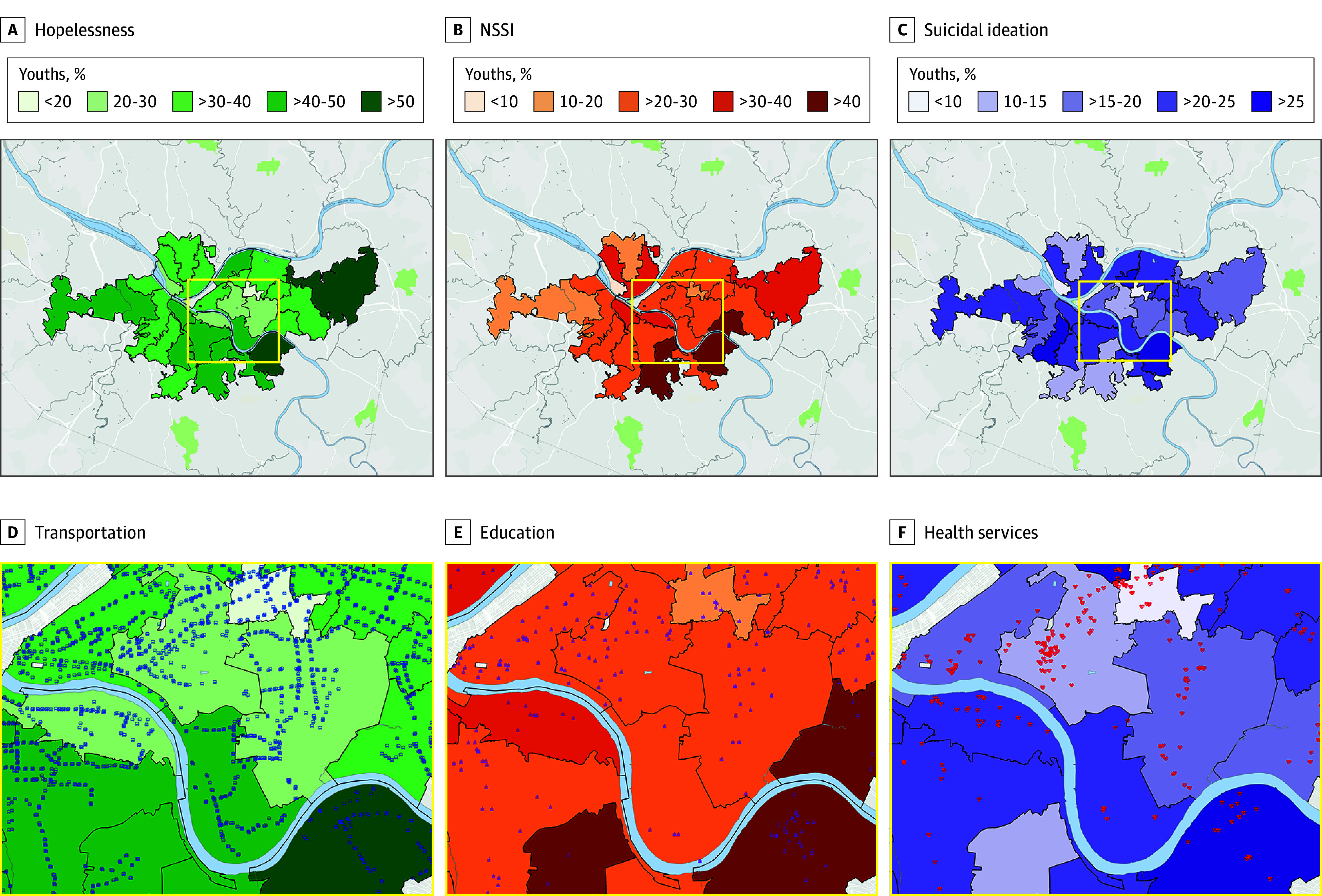
Mental Health and Community Assets in Allegheny County, Pennsylvania Choropleth maps show percentages of youths reporting past-year hopelessness (A), nonsuicidal self-injury (NSSI) (B), and suicidal ideation (C) across 26 zip codes in Allegheny County, Pennsylvania. Insets show community asset locations for transportation (D), educational resources (E), and health services (F). Data are derived from the results of the 2018 Allegheny County Youth Risk Behavior Survey.

**Table 2.** Associations Between Community-Level Asset Density and Mental Health^a^

Asset category	Mental health measure					
	Hopelessness		NSSI		Suicidal ideation	
	AOR (95% CI)	*P* value	AOR (95% CI)	*P* value	AOR (95% CI)	*P* value
**Transportation**						
Population density	0.77 (0.66-0.90)	<.001	1.02 (0.87-1.21)	.80	0.87 (0.72-1.04)	.13
Spatial density	0.83 (0.73-0.95)	.006	0.97 (0.84-1.12)	.68	0.97 (0.83-1.14)	.71
**Education**						
Population density	0.78 (0.67-0.92)	.002	1.00 (0.84-1.18)	.97	0.88 (0.74-1.06)	.18
Spatial density	0.87 (0.77-0.98)	.02	0.97 (0.85-1.11)	.69	0.99 (0.86-1.14)	.91
**Parks and recreation**						
Population density	0.86 (0.74-1.01)	.06	1.05 (0.89-1.24)	.57	0.93 (0.78-1.12)	.46
Spatial density	0.90 (0.78-1.04)	.15	1.01 (0.87-1.18)	.89	1.02 (0.87-1.20)	.81
**Faith-based entities**						
Population density	0.85 (0.73-0.99)	.04	1.10 (0.93-1.29)	.26	0.94 (0.78-1.12)	.47
Spatial density	0.93 (0.84-1.04)	.22	1.02 (0.91-1.16)	.70	1.03 (0.90-1.17)	.65
**Health services**						
Population density	0.74 (0.60-0.91)	.006	1.03 (0.85-1.25)	.77	0.85 (0.67-1.08)	.19
Spatial density	0.80 (0.67-0.95)	.01	1.01 (0.84-1.20)	.95	0.94 (0.77-1.15)	.54
**Food resources**						
Population density	0.90 (0.79-1.02)	.11	0.95 (0.82-1.10)	.48	0.98 (0.84-1.14)	.80
Spatial density	0.89 (0.81-0.98)	.02	0.95 (0.85-1.06)	.39	1.02 (0.90-1.14)	.77
** Personal care services**						
Population density	0.97 (0.85-1.11)	.65	0.96 (0.83-1.11)	.57	0.99 (0.85-1.16)	.92
Spatial density	0.92 (0.78-1.09)	.35	1.00 (0.83-1.19)	.96	1.05 (0.87-1.27)	.60
**Social infrastructure**						
Population density	0.93 (0.85-1.02)	.14	1.04 (0.94-1.15)	.44	0.97 (0.87-1.08)	.62
Spatial density	0.94 (0.86-1.03)	.16	1.04 (0.95-1.13)	.44	0.99 (0.90-1.09)	.83
**Total assets**						
Population density	0.85 (0.75-0.97)	.01	1.05 (0.92-1.21)	.45	0.93 (0.80-1.08)	.34
Spatial density	0.87 (0.78-0.98)	.02	1.04 (0.91-1.18)	.56	0.98 (0.86-1.13)	.82

Abbreviations: AOR, adjusted odds ratio; NSSI, nonsuicidal self-injury.

^a^ Generalized linear mixed models were adjusted for age, sex assigned at birth, race and ethnicity, identification as sexually or gender diverse, Child Opportunity Index score (national norm; scores range from 1 to 100 based on percentile rank, with higher scores indicating more favorable opportunity), and school-level clustering.

In models examining COI scores, the overall COI score was significantly associated with lower odds of hopelessness (AOR, 0.89 [95% CI, 0.81-0.97]). A similar association was noted for health and environment (AOR, 0.90 [95% CI, 0.82-0.99]) and social and economic (AOR, 0.88 [95% CI, 0.81-0.96]) domains of the COI but not for education (AOR, 0.92 [95% CI, 0.83-1.01]) (Table 3). There were no correlations among asset density, COI, and other mental health measures. Total asset density did not show significant correlations with overall COI score (*r* = −0.26 [95% CI, −0.59 to 0.19]; *P* = .19).

**Table 3.** Associations Between Locally Normalized Child Opportunity Index and Mental Health^a^

COI domain^b^	Mental health measure					
	Hopelessness		NSSI		Suicidal ideation	
	AOR (95% CI)	*P* value	AOR (95% CI)	*P* value	AOR (95% CI)	*P* value
Education	0.92 (0.83-1.01)	.09	1.00 (0.89-1.11)	.95	0.97 (0.86-1.10)	.65
Health and environment	0.90 (0.82-0.99)	.02	0.97 (0.88-1.08)	.60	0.92 (0.82-1.03)	.15
Social and economic	0.88 (0.81-0.96)	.003	0.97 (0.89-1.07)	.57	0.93 (0.84-1.03)	.19
Overall	0.89 (0.81-0.97)	.006	0.98 (0.89-1.07)	.65	0.94 (0.84-1.04)	.21

Abbreviations: AOR, adjusted odds ratio; COI, Child Opportunity Index; NSSI, nonsuicidal self-injury.

^a^ Generalized linear mixed models were adjusted for age, sex assigned at birth, race and ethnicity, identification as sexually or gender diverse, and school-level clustering.

^b^ Scores range from 1 to 100 based on percentile rank, with higher scores indicating more favorable opportunity.

## Discussion

In this cross-sectional study that leveraged a unique county-specific geospatial dataset, we demonstrated significant associations between community assets and youths’ mental health. We found that youths living in zip codes with a high density of transportation assets (eg, bus stops, light-rail stations), educational centers (eg, schools, colleges), faith-based entities (churches, synagogues, or temples), health services (eg, hospitals, doctors’ offices), and food resources (eg, grocery stores, food banks) reported lower odds of hopelessness. These findings were supported using a census-derived COI score. Our study builds on existing work^9,10,12,13,14,15,16,17,18,19,37,38^ by incorporating innovative quantitative methods and exploring a wide range of community assets. In addition, the specificity of data in this study allowed for a more nuanced understanding of the associations among spatial factors, resource access, and mental health.

The association between health service density and hopelessness among youths builds on existing geospatial research involving mental health care access. One prior study in an adult sample suggested that proximity to a primary care office was associated with depressive symptoms.^39^ That said, most other studies in this domain have focused on operationalizing measures of spatial access (eg, transit time) or describing specific access determinants (eg, proximity to transportation)^40,41,42^ rather than exploring associations with mental health symptoms. Although we did not analyze youths’ actual service utilization, the observed correlation may support theories of early service access associated with reduced mental health morbidity.^43^ This notion may be further supported by the inverse associations noted between transportation-related assets and hopelessness in our study. A high density of health clinics may also indicate greater clinician availability, a known facilitator of mental health care engagement among young people.^44^

In terms of other community assets, our findings regarding food resources support prior work, which has shown that food security may predict better mental health.^45,46,47^ Educational assets, such as high schools and colleges, showed similar negative associations with hopelessness, which may indicate the role of embedded mental health supports (eg, school counselors), classroom size, or other elements of school climate in promoting positive psychologic outcomes.^48,49^ In contrast, we noted no associations between the presence of parks and recreational spaces with the mental health measures examined. This may be due to the structure of our assets’ database. Each asset was coded as a point location (latitude and longitude) and thus did not capture areal factors, such as the spatial proportion of each zip code covered in green space.

Notably, significant associations were observed for hopelessness but not other mental health measures (ie, NSSI or suicidal ideation). A majority of geospatial studies involving youth mental health have focused on suicidal ideation, self-injury, or suicide attempts, with fewer considering other depressive symptoms.^50,51,52^ This is an important distinction, as increasing hope and goals for the future (ie, future orientation) has emerged as a cross-cutting protective factor for multiple adolescent health outcomes, especially among youths living in areas with a concentrated disadvantage.^53,54,55^ Among previous studies that have specifically considered hopelessness, the findings have generally supported the role of certain aspects of the built environment, such as housing occupancy and physical disorder (eg, graffiti, litter, or broken glass), as environmental determinants of these symptoms.^56,57,58^ That said, such studies have primarily assessed adult populations, and a majority have focused on census-based indicators included in composite deprivation indices such as the COI. There is a relative dearth in research examining community-protective factors and hopelessness, and our analyses suggest that certain assets may interact independently with youth mental health even after controlling for differences in neighborhood context.

It is important to note that both COI and asset density do not measure all forms of social, economic, and cultural capital in a neighborhood. For example, there were 2 zip codes with relatively low asset density that exhibited lower rates of hopelessness than what would be expected based on anticipated associations (eFigure 3 in Supplement 1). These neighborhoods have been disproportionately impacted by historical forms of oppression, which may be reflected as poorer scores on measures of deprivation (ie, low COI); however, findings of low hopelessness indicated that youths in such neighborhoods may have been buffered against negative mental health outcomes due to other unmeasured resources in the area. In addition, asset density did not show apparent correlations with COI, further distinguishing community assets as a measure that does not solely represent the absence of deprivation. Future work must involve partnerships with community members to comprehensively characterize neighborhood-level assets.^59^

### Strengths and Limitations

Notable strengths of our study include the specificity offered by our measurement of community assets and the broad range of asset categories—features that complement existing measures of community opportunity. Our sample included youths with diverse racial, ethnic, sexual, and gender identities, which supports the generalizability of our findings. Because our study leveraged a unique locally derived dataset, we have been able to share findings with community collaborators to foster data transparency and promote translation of research findings. Obtaining additional qualitative data from youths through participatory mapping^60^ and walking interviews^45,46^ may further illuminate assets that support youths’ mental health. Focus groups or surveys with community members may allow identification of other assets not represented in existing repositories. Linking asset data to mental health measures collected at the census tract level may offer insight into neighborhood variability not apparent at the zip code level. Sharing data with policy makers and funders may inform structural interventions that fortify existing bright spots.

This study also has several key limitations. Our analysis was limited by geocoding at the zip code level, which may have obscured important heterogeneity within zip codes. In addition, zip codes are comprised of addresses defined yearly by the US Postal Service and do not represent distinct geographic boundaries, which creates challenges related to data aggregation.^61,62^ Although our study considered over 20 000 community assets, the specific services provided by each resource (eg, whether they offer youth-serving programs) were not included in the repository, making it difficult to quantify reach or associations with these locations. The YRBS was administered to students who were enrolled in urban high schools, which may cause sampling bias by excluding youths in rural areas or those with chronic absenteeism. Furthermore, our study only included youths from 26 of over 100 zip codes in Allegheny County, which may be due, in part, to a large percentage of missing zip code data in the overall YRBS dataset. Youths with missing zip code data were more likely to be younger, male, and Black, highlighting the need for more focused efforts to understand geospatial context in these subgroups.

In this study, we relied on cross-sectional data collected at multiple points in time. The WPRDC asset database was compiled between 2019 and 2020, the year after the YRBS survey was administered, and while most assets demonstrated relative stability prior to the COVID-19 pandemic, there were likely more prominent shifts after March 1, 2020. The COI data were derived from census-based indicators collected in 2015, which predates both the YRBS and WPRDC data. Because of these challenges, we could not examine longitudinal trends in asset density or mental health measures, and inferences related to causation were also limited. That said, many elements of neighborhood context, such as transportation, safety, and educational opportunity, do not change rapidly due to long-standing histories of structural oppression, reinforcing the critical importance of identifying and elevating community-level protective factors.

## Conclusions

Methods to understand and analyze community assets through geospatial mapping continue to evolve. This cross-sectional study highlights opportunities to integrate strength-based frameworks in describing neighborhood context and suggests that certain asset types may be particularly relevant for addressing youths’ mental health. Future work must synergize existing data sources with expertise from community members to collect additional spatial metrics, implement novel analytic approaches, and develop interventions that fortify neighborhood resources to support youths in the areas in which they live and play.

## Notes

Supplement 1. **eTable 1.** Data Sources and Measures **eFigure 1.** Study Flow Diagram **eTable 2.** Comparison of Youth With and Without Missing Zip Code Data **eTable 3.** Asset Categories Derived From Allegheny County Assets Database **eFigure 2.** Multivariate Cluster Analysis of Mental Health Measures **eTable 4.** Associations Between Community-Level Asset Density and Mental Health: Unadjusted Odds Ratios (95% CIs) **eTable 5.** Associations between Community-Level Asset Density and Mental Health: Adjusted Odds Ratios (95% CIs) **eFigure 3.** “Bright Spot” Analysis: Asset Density Versus Proportion of Youth Reporting Hopelessness

Supplement 2.
Data Sharing Statement

## References

[1] Youth risk behavior survey: data summary & trends report 2013. Centers for Disease Control and Prevention. Accessed August 7, 2024. https://www.cdc.gov/yrbs/dstr/index.html

[2] Duke NN, Borowsky IW, Pettingell SL, McMorris BJ. Examining youth hopelessness as an independent risk correlate for adolescent delinquency and violence. Matern Child Health J. 2011;15(1):87-97. doi:10.1007/s10995-009-0550-6 20012345

[3] Cipriano A, Cella S, Cotrufo P. Nonsuicidal self-injury: a systematic review. Front Psychol. 2017;8(NOV):1946. doi:10.3389/fpsyg.2017.01946 29167651 PMC5682335

[4] Thapar A, Eyre O, Patel V, Brent D. Depression in young people. Lancet. 2022;400(10352):617-631. doi:10.1016/S0140-6736(22)01012-1 35940184

[5] Stirling K, Toumbourou JW, Rowland B. Community factors influencing child and adolescent depression: a systematic review and meta-analysis. Aust N Z J Psychiatry. 2015;49(10):869-886. doi:10.1177/0004867415603129 26416916

[6] Hoffmann JA, Farrell CA, Monuteaux MC, Fleegler EW, Lee LK. Association of pediatric suicide with county-level poverty in the United States, 2007-2016. JAMA Pediatr. 2020;174(3):287-294. doi:10.1001/jamapediatrics.2019.5678 31985759 PMC6990805

[7] Bonnie RJ, Backes EP, eds. The Promise of Adolescence: Realizing Opportunity for All Youth. National Academies Press; 2019. doi:10.17226/25388 31449373

[8] Weinstein JN, Geller A, Negussie Y, Baciu A, eds. Communities in Action: Pathways to Health Equity. National Academies Press; 2017. doi:10.17226/24624 28418632

[9] Theokas C, Lerner RM. Observed ecological assets in families, schools, and neighborhoods: conceptualization, measurement, and relations with positive and negative developmental outcomes. Appl Dev Sci. 2006;10(2):61-74. doi:10.1207/s1532480xads1002_2

[10] Urban JB, Lewin-Bizan S, Lerner RM. The role of neighborhood ecological assets and activity involvement in youth developmental outcomes: differential impacts of asset poor and asset rich neighborhoods. J Appl Dev Psychol. 2009;30(5):601-614. doi:10.1016/j.appdev.2009.07.003

[11] Park YS, McMorris BJ, Pruinelli L, Song Y, Kaas MJ, Wyman JF. Use of geographic information systems to explore associations between neighborhood attributes and mental health outcomes in adults: a systematic review. Int J Environ Res Public Health. 2021;18(16):8597. doi:10.3390/ijerph18168597 34444345 PMC8393279

[12] Alaimo K, Beavers AW, Crawford C, Snyder EH, Litt JS. Amplifying health through community gardens: a framework for advancing multicomponent, behaviorally based neighborhood interventions. Curr Environ Health Rep. 2016;3(3):302-312. doi:10.1007/s40572-016-0105-0 27379424

[13] Booth JM, Chapman D, Ohmer ML, Wei K. Examining the relationship between level of participation in community gardens and their multiple functions. J Community Pract. 2018;26(1):5-22. doi:10.1080/10705422.2017.1413024

[14] Zhang J, Yu Z, Zhao B, Sun R, Vejre H. Links between green space and public health: a bibliometric review of global research trends and future prospects from 1901 to 2019. Environ Res Lett. 2020;15(6):063001. doi:10.1088/1748-9326/ab7f64

[15] Dzhambov AM, Markevych I, Hartig T, . Multiple pathways link urban green- and bluespace to mental health in young adults. Environ Res. 2018;166:223-233. doi:10.1016/j.envres.2018.06.004 29890427

[16] Tillmann S, Tobin D, Avison W, Gilliland J. Mental health benefits of interactions with nature in children and teenagers: a systematic review. J Epidemiol Community Health. 2018;72(10):958-966. doi:10.1136/jech-2018-210436 29950520 PMC6161651

[17] Philbin MM, Parker CM, Flaherty MG, Hirsch JS. Public libraries: a community-level resource to advance population health. J Community Health. 2019;44(1):192-199. doi:10.1007/s10900-018-0547-4 29995303 PMC6329675

[18] Gelzhiser JA, Lewis L. Black barbers as mental health advocates, and interpersonal violence and suicide preventors in the local community. Ment Health Prev. 2023;31:200291. doi:10.1016/j.mhp.2023.200291

[19] Palmer KNB, Rivers PS, Melton FL, . Health promotion interventions for African Americans delivered in U.S. barbershops and hair salons—a systematic review. BMC Public Health. 2021;21(1):1553. doi:10.1186/s12889-021-11584-0 34399723 PMC8365990

[20] Afulani PA, Coleman-Jensen A, Herman D. Food insecurity, mental health, and use of mental health services among nonelderly adults in the United States. J Hunger Environ Nutr. 2020;15(1):29-50. doi:10.1080/19320248.2018.1537868

[21] Tarasuk V, Cheng J, Gundersen C, de Oliveira C, Kurdyak P. The relation between food insecurity and mental health care service utilization in Ontario. Can J Psychiatry. 2018;63(8):557-569. doi:10.1177/0706743717752879 29307216 PMC6099753

[22] Kyle T, Dunn JR. Effects of housing circumstances on health, quality of life and healthcare use for people with severe mental illness: a review. Health Soc Care Community. 2008;16(1):1-15. doi:10.1111/j.1365-2524.2007.00723.x 18181811

[23] Musa GJ, Chiang PH, Sylk T, . Use of GIS mapping as a public health tool—from cholera to cancer. Health Serv Insights. 2013;6:111-116. doi:10.4137/HSI.S10471 25114567 PMC4089751

[24] Wang F. Why public health needs GIS: a methodological overview. Ann GIS. 2020;26(1):1-12. doi:10.1080/19475683.2019.1702099 32547679 PMC7297184

[25] Zhu L, Gorman DM, Horel S. Alcohol outlet density and violence: a geospatial analysis. Alcohol. 2004;39(4):369-375. doi:10.1093/alcalc/agh062 15208173

[26] Reitzel LR, Regan SD, Nguyen N, . Density and proximity of fast food restaurants and body mass index among African Americans. Am J Public Health. 2014;104(1):110-116. doi:10.2105/AJPH.2012.301140 23678913 PMC3910025

[27] Trivedi M, Beck AF, Garg A. Bringing geospatial awareness to community pediatrics and primary care. Pediatrics. 2022;149(4):e2021053926. doi:10.1542/peds.2021-053926 35362063 PMC9647568

[28] Acevedo-Garcia D, McArdle N, Hardy EF, . The child opportunity index: improving collaboration between community development and public health. Health Aff (Millwood). 2014;33(11):1948-1957. doi:10.1377/hlthaff.2014.0679 25367989

[29] Butler DC, Petterson S, Phillips RL, Bazemore AW. Measures of social deprivation that predict health care access and need within a rational area of primary care service delivery. Health Serv Res. 2013;48(2, Pt 1):539-559. doi:10.1111/j.1475-6773.2012.01449.x 22816561 PMC3626349

[30] Trinidad S, Brokamp C, Mor Huertas A, . Use of area-based socioeconomic deprivation indices: a scoping review and qualitative analysis. Health Aff (Millwood). 2022;41(12):1804-1811. doi:10.1377/hlthaff.2022.00482 36469826

[31] Reiss F. Socioeconomic inequalities and mental health problems in children and adolescents: a systematic review. Soc Sci Med. 2013;90:24-31. doi:10.1016/j.socscimed.2013.04.026 23746605

[32] Visser K, Bolt G, Finkenauer C, Jonker M, Weinberg D, Stevens GWJM. Neighbourhood deprivation effects on young people’s mental health and well-being: a systematic review of the literature. Soc Sci Med. 2021;270:113542. doi:10.1016/j.socscimed.2020.113542 33495056

[33] Datasets. Western Pennsylvania Regional Data Center. Accessed August 1, 2023. https://data.wprdc.org/dataset

[34] Child Opportunity Index 2.0 ZIP Code data. The Heller School for Social Policy and Management. Accessed August 1, 2023. https://data.diversitydatakids.org/dataset/coi20_zipcodes-child-opportunity-index-2-0-zip-code-data

[35] Kidd KM, Sequeira GM, Rothenberger SD, . Operationalizing and analyzing 2-step gender identity questions: methodological and ethical considerations. J Am Med Inform Assoc. 2022;29(2):249-256. doi:10.1093/jamia/ocab137 34472616 PMC8757306

[36] Szoko N, Sequeira GM, Coulter RWS, . Sexual orientation among gender diverse youth. J Adolesc Health. 2023;72(1):153-155. doi:10.1016/j.jadohealth.2022.08.016 36216680 PMC10748722

[37] Whatley E, Fortune T, Williams AE. Enabling occupational participation and social inclusion for people recovering from mental ill-health through community gardening. Aust Occup Ther J. 2015;62(6):428-437. doi:10.1111/1440-1630.12240 26530278

[38] Culyba AJ, Jacoby SF, Richmond TS, Fein JA, Hohl BC, Branas CC. Modifiable neighborhood features associated with adolescent homicide. JAMA Pediatr. 2016;170(5):473-480. doi:10.1001/jamapediatrics.2015.4697 26954939 PMC4936414

[39] Tomita A, Vandormael AM, Cuadros D, Slotow R, Tanser F, Burns JK. Proximity to healthcare clinic and depression risk in South Africa: geospatial evidence from a nationally representative longitudinal study. Soc Psychiatry Psychiatr Epidemiol. 2017;52(8):1023-1030. doi:10.1007/s00127-017-1369-x 28299376 PMC5534383

[40] Wang F. Measurement, optimization, and impact of health care accessibility: a methodological review. Ann Assoc Am Geogr. 2012;102(5):1104-1112. doi:10.1080/00045608.2012.657146 23335813 PMC3547595

[41] Higgs G. A literature review of the use of GIS-based measures of access to health care services. Health Serv Outcomes Res Methodol. 2004;5(2):119-139. doi:10.1007/s10742-005-4304-7

[42] Smith-East M, Neff DF. Mental health care access using geographic information systems: an integrative review. Issues Ment Health Nurs. 2020;41(2):113-121. doi:10.1080/01612840.2019.1646363 31661647

[43] McGorry PD, Mei C. Early intervention in youth mental health: progress and future directions. Evid Based Ment Health. 2018;21(4):182-184. doi:10.1136/ebmental-2018-300060 30352884 PMC10270418

[44] Radez J, Reardon T, Creswell C, Lawrence PJ, Evdoka-Burton G, Waite P. Why do children and adolescents (not) seek and access professional help for their mental health problems? a systematic review of quantitative and qualitative studies. Eur Child Adolesc Psychiatry. 2021;30(2):183-211. doi:10.1007/s00787-019-01469-4 31965309 PMC7932953

[45] Compton MT, Ku BS. Prevalence of food insecurity and living in a food desert among individuals with serious mental illnesses in public mental health clinics. Community Ment Health J. 2023;59(2):357-362. doi:10.1007/s10597-022-01013-w 35963919 PMC10209833

[46] Nagata JM, Ganson KT, Whittle HJ, . Food insufficiency and mental health in the U.S. during the COVID-19 pandemic. Am J Prev Med. 2021;60(4):453-461. doi:10.1016/j.amepre.2020.12.004 33602534 PMC9067067

[47] Fang D, Thomsen MR, Nayga RM Jr. The association between food insecurity and mental health during the COVID-19 pandemic. BMC Public Health. 2021;21(1):607. doi:10.1186/s12889-021-10631-0 33781232 PMC8006138

[48] Wang MT, Degol JL. School climate: a review of the construct, measurement, and impact on student outcomes. Educ Psychol Rev. 2016;28(2):315-352. doi:10.1007/s10648-015-9319-1

[49] Aldridge JM, McChesney K. The relationships between school climate and adolescent mental health and wellbeing: a systematic literature review. Int J Educ Res. 2018;88:121-145. doi:10.1016/j.ijer.2018.01.012

[50] Sugg MM, Runkle JD, Andersen LM, Desjardins MR. Exploring place-based differences in suicide and suicide-related outcomes among North Carolina adolescents and young adults. J Adolesc Health. 2023;72(1):27-35. doi:10.1016/j.jadohealth.2022.06.013 35985915

[51] Froberg BA, Morton SJ, Mowry JB, Rusyniak DE. Temporal and geospatial trends of adolescent intentional overdoses with suspected suicidal intent reported to a state poison control center. Clin Toxicol (Phila). 2019;57(9):798-805. doi:10.1080/15563650.2018.1554186 30696297

[52] Hill RM, Gushanas KL, Alvis L, . Geospatial identification of high youth suicide risk areas via electronic health records: avenues for research and prevention efforts. Suicide Life Threat Behav. 2021;51(2):255-262. doi:10.1111/sltb.12701 33876482

[53] Johnson SR, Blum RW, Cheng TL. Future orientation: a construct with implications for adolescent health and wellbeing. Int J Adolesc Med Health. 2014;26(4):459-468. doi:10.1515/ijamh-2013-0333 24523304 PMC4827712

[54] So S, Voisin DR, Burnside A, Gaylord-Harden NK. Future orientation and health related factors among African American adolescents. Child Youth Serv Rev. 2016;61:15-21. doi:10.1016/j.childyouth.2015.11.026

[55] Khetarpal SK, Jeong K, Abebe KZ, Miller E, Culyba AJ. Examining longitudinal associations between future orientation and multiple forms of youth violence perpetration. J Adolesc Health. 2023;73(1):95-101. doi:10.1016/j.jadohealth.2023.01.027 36914448 PMC10846914

[56] Mair C, Diez Roux AV, Galea S. Are neighbourhood characteristics associated with depressive symptoms? a review of evidence. J Epidemiol Community Health. 2008;62(11):940-946. doi:10.1136/jech.2007.066605 18775943

[57] Mair C, Kaplan GA, Everson-Rose SA. Are there hopeless neighborhoods? an exploration of environmental associations between individual-level feelings of hopelessness and neighborhood characteristics. Health Place. 2012;18(2):434-439. doi:10.1016/j.healthplace.2011.12.012 22265206 PMC3285550

[58] Snedker KA, Herting JR. Adolescent mental health: neighborhood stress and emotional distress. Youth Soc. 2013;48(5):695-719. doi:10.1177/0044118X13512335

[59] Fagerholm N, Raymond CM, Olafsson AS, . A methodological framework for analysis of participatory mapping data in research, planning, and management. Int J Geogr Inf Sci. 2021;35(9):1848-1875. doi:10.1080/13658816.2020.1869747

[60] Literat I. Participatory mapping with urban youth: the visual elicitation of socio-spatial research data. Learn Media Technol. 2013;38(2):198–216. doi:10.1080/17439884.2013.782037

[61] Grubesic TH. Zip codes and spatial analysis: problems and prospects. Socioecon Plann Sci. 2008;42(2):129-149. doi:10.1016/j.seps.2006.09.001

[62] Grubesic TH, Matisziw TC. On the use of ZIP codes and ZIP code tabulation areas (ZCTAs) for the spatial analysis of epidemiological data. Int J Health Geogr. 2006;5(1):58. doi:10.1186/1476-072X-5-58 17166283 PMC1762013

