# Supplementary material for: Community Asset Density and Past-Year Mental Health Symptoms Among Youths
Source: JAMA Netw Open. 2024 Sep 20;7(9):e2434923. doi: 10.1001/jamanetworkopen.2024.34923 (PMC11415787; doi:10.1001/jamanetworkopen.2024.34923)
Supplement: Supplement 1. — eTable 1. Data Sources and Measures eFigure 1. Study Flow Diagram eTable 2. Comparison of Youth With and Without Missing Zip Code Data eTable 3. Asset Categories Derived From Allegheny County Assets Database eFigure 2. Multivariate Cluster Analysis of Mental Health Measures eTable 4. Associations Between Community-Level Asset Density and Mental Health: Unadjusted Odds Ratios (95% CIs) eTable 5. Associations between Community-Level Asset Density and Mental Health: Adjusted Odds Ratios (95% CIs) eFigure 3. “Bright Spot” Analysis: Asset Density Versus Proportion of Youth Reporting Hopelessness [file jamanetwopen-e2434923-s001.pdf]

Supplemental Online Content

Szoko N, Ajith A, Kurland K, Culyba AJ. Community asset density and past-year mental health symptoms among youths. *JAMA Netw Open*. 2024;7(9):e2434923.
doi:10.1001/jamanetworkopen.2024.34923

eTable 1. Data Sources and Measures
eFigure 1. Study Flow Diagram
eTable 2. Comparison of Youth With and Without Missing Zip Code Data
eTable 3. Asset Categories Derived From Allegheny County Assets Database
eFigure 2. Multivariate Cluster Analysis of Mental Health Measures
eTable 4. Associations Between Community-Level Asset Density and Mental Health: Unadjusted Odds Ratios (95% CIs)
eTable 5. Associations between Community-Level Asset Density and Mental Health: Adjusted Odds Ratios (95% CIs)
eFigure 3. “Bright Spot” Analysis: Asset Density Versus Proportion of Youth Reporting Hopelessness

This supplemental material has been provided by the authors to give readers additional information about their work.

© 2024 Szoko N et al. *JAMA Network Open*.

eTable 1. Data Sources and Measures

Dataset	Construct	Item / Measure	Operationalization
Allegheny County Youth Risk Behavior Survey[a]	Demographics		
	Age	How old are you?	Age in years (continuous)
	Sex	What is your sex (the sex you were assigned at birth, on your birth certificate)?	Female/male (binary)
	Race	What is your race? (select one or more responses): American Indian or Alaska Native, Asian, Black or African American, Native Hawaiian or Other Pacific Islander, White, Other	Collapsed race/ethnicity variable with three categories: non-Hispanic White, non-Hispanic Black, Hispanic/Multiracial/Other (categorical)
	Ethnicity	Are you Hispanic/Latino?	
	Gender Identity	Which of the following best describes your gender identity? (select all that apply): girl, boy, trans girl, trans boy, genderqueer, non-binary, another identity	Youth reporting any sexual identity other than heterosexual or any gender identity other than cisgender (binary)
	Sexual Identity	Which of the following best describes you? (select all that apply): heterosexual, mostly heterosexual, gay/lesbian, bisexual, queer, asexual, not sure	
	Mental Health Measures		
	Hopelessness	During the past 12 months, did you ever feel so sad or hopeless almost every day for two weeks or more in a row that you stopped doing some usual activities?	Yes/no (binary)
	Non-Suicidal Self-Injury	During the past 12 months, how many times have you ever hurt yourself on purpose without wanting to die, such as cutting, pinching, scratching, or burning yourself?	Frequency operationalized to any/none (binary)
	Suicidal Ideation	During the past 12 months, did you ever seriously consider attempting suicide?	Yes/no (binary)
Allegheny County Assets[b]	Asset Density	Point (latitude/longitude) data for 32,938 assets; 23,611 assets were coded into eight categories based on review of literature and *a priori* hypotheses (see Supplement 3)	Calculated total number of assets per zip code and divided by either 1) zip code area (spatial density) or 2) population under age 18 in that zip code (population density); normalized to dataset with z-score
Child Opportunity Index 2.0[c]	COI Score	Zip code level COI score aggregated from >72,000 census tracts (range: 1-100); includes 19 indicators across three categories: Education, Health & Environment, Social & Economic	Normalized to dataset with z-score
	Population under age 18	Total estimated population under 18 derived from 5-year American Community Survey (ACS) data	Total estimated population under 18 residing in each zip code

a. Allegheny County Health Department. Youth Risk Behavior Survey (2018). Dataset maintained by Dr. Elizabeth Miller, MD, PhD, University of Pittsburgh.
b. Western Pennsylvania Regional Data Center. *Allegheny County Assets.* Web. 14 April 2023. https://data.wprdc.org/dataset/allegheny-county-assets
c. diversitydatakids.org. *Child Opportunity Index 2.0 ZIP Code data*. Web. 7 Aug 2023. https://data.diversitydatakids.org/dataset/coi20_zipcodes-child-opportunity-index-2-0-zip-code-data?_external=True

© 2024 Szoko N et al. *JAMA Network Open*.

eFigure 1. Study Flow Diagram

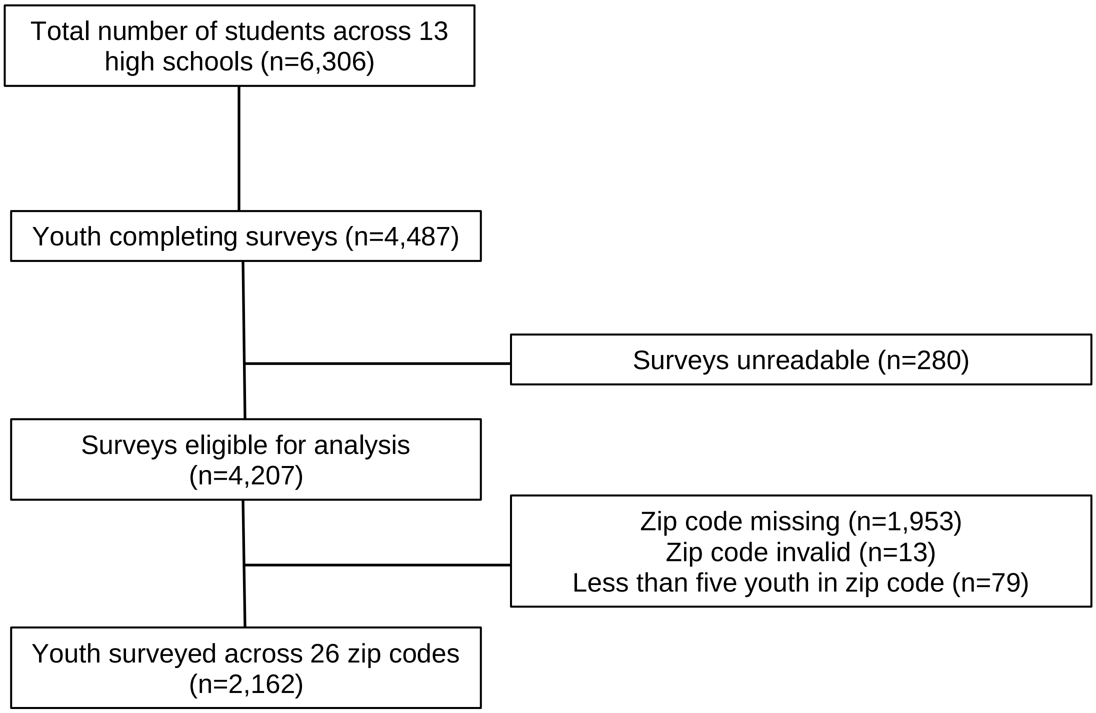

© 2024 Szoko N et al. *JAMA Network Open*.

eTable 2. Comparison of Youth With and Without Missing Zip Code Data (n = 4207)

Characteristic	Not missing	Missing	*P* value[a]
**Age:** mean (SD)	**15.8 (1.2)**	**15.6 (1.3)**	**<0.001**
**Sex** (Female)	**1276 (57.5)**	**942 (42.5)**	**<0.001**
**Race/Ethnicity**			**<0.001**
White	**1011 (71.1)**	**411 (28.9)**	
Black	**591 (40.1)**	**884 (59.9)**	
Other	**609 (50.3)**	**601 (49.7)**	
**Identification as Sexual/Gender Minority**	**589 (63.3)**	**342 (36.7)**	**0.08**
**Parental Education**			**<0.001**
Some High School	**391 (69.9)**	**168 (30.1)**	
Graduated High School	**1572 (83.9)**	**302 (16.1)**	
**Hopelessness**	**843 (57.0)**	**635 (43.0)**	**0.531**
**NSSI**	**617 (60.6)**	**401 (39.4)**	**0.001**
**Suicidal Ideation**	**475 (60.1)**	**316 (39.9)**	**0.029**

[a]*P* value derived from Kruskal Wallis or chi-squared test for continuous and categorical variables, respectively.

© 2024 Szoko N et al. *JAMA Network Open*.

eTable 3. Asset Categories Derived From Allegheny County Assets Database[a]

Asset Category	No. (%) N=32,938
**Category A: Transportation**	
Bike share stations	113 (0.3)
Bus stops	6,752 (20.5)
Park and rides	52 (0.2)
**Category B: Education**	
Childcare centers	646 (2)
Schools	1,013 (3.1)
Universities	54 (0.2)
**Category C: Parks and Recreation**	
Parks and facilities	827 (2.5)
Recreation centers	21 (0.1)
**Category D: Faith-Based**	
Faith-based facilities	1,197 (3.6)
**Category E: Health Services**	
Allegheny County Health Department clinics	16 (0)
Dentists	285 (0.9)
Doctors' offices	607 (1.8)
Health centers	256 (0.8)
Pharmacies	331 (1.0)
Women, Infants, and Children (WIC) offices	11 (0)
**Category F: Food Resources**	
WIC vendors	97 (0.3)
Farmers markets	91 (0.3)
Food banks	77 (0.2)
Supermarkets	133 (0.4)
**Category G: Personal Care Services**	
Barbers	214 (0.6)
Hair salons	1,060 (3.2)
Nail salons	257 (0.8)
**Category H: Social Infrastructure**	
Family support centers	30 (0.1)
Community / nonprofit organizations	9,325 (28.3)
Libraries	91 (0.3)
Museums	44 (0.1)
**Other[b]**	9,338 (28.4)

a. Derived from Western Pennsylvania Regional Data Center (https://data.wprdc.org/dataset).
b. Other assets included commercial/retail spaces (e.g., coffee shops, restaurants), housing facilities (e.g., apartment buildings, hotels), and certain governmental locations (e.g., post offices, polling places).

© 2024 Szoko N et al. *JAMA Network Open*.

eFigure 2. Multivariate Cluster Analysis of Mental Health Measures

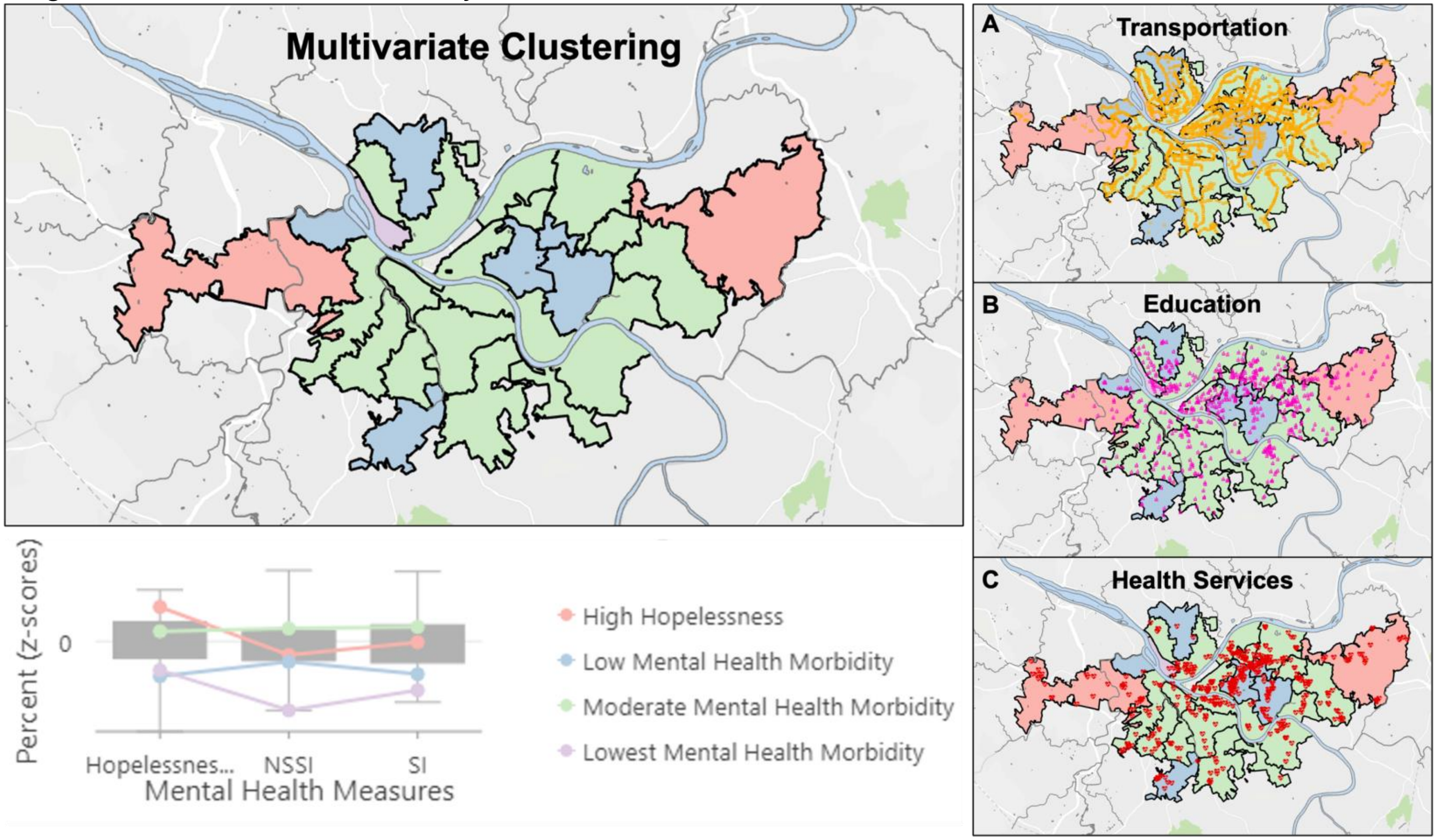

Legend (eFigure 2): We conducted multivariate clustering analysis (k-means) on standardized (z-score) percentages of youth reporting mental health concerns in each zip code. We reviewed 2, 3, 4, and 5 cluster solutions; a 4-cluster solution was selected based on differentiation and overall fit. eFigure 2 displays 4 predominant clusters: high hopelessness, moderate mental health morbidity, low mental health morbidity, and lowest mental health morbidity. Panels A, B, and C show maps overlayed with three asset types (Transportation, Education, and Health Services), demonstrating that areas with high levels of hopelessness appear to have a lower density of these asset categories, aligning with what we present in our primary manuscript.

© 2024 Szoko N et al. *JAMA Network Open*.

eTable 4. Associations Between Community-Level Asset Density and Mental Health: Unadjusted Odds Ratios (95% CIs)[a]

Asset Category	Hopelessness	Non-Suicidal Self-Injury	Suicidal Ideation
**Category A: Transportation**			
Population Density	0.89 (0.78-1.02)	1.00 (0.86-1.16)	0.93 (0.79-1.09)
Spatial Density	0.92 (0.81-1.03)	0.96 (0.84-1.09)	0.99 (0.86-1.14)
**Category B: Education**			
Population Density	0.90 (0.79-1.03)	0.96 (0.83-1.11)	0.95 (0.81-1.11)
Spatial Density	0.95 (0.86-1.06)	0.95 (0.85-1.07)	1.02 (0.90-1.15)
**Category C: Parks and Recreation**			
Population Density	0.99 (0.87-1.14)	1.05 (0.91-1.21)	0.99 (0.84-1.16)
Spatial Density	1.02 (0.91-1.15)	1.02 (0.90-1.16)	1.05 (0.92-1.21)
**Category D: Faith-Based**			
Population Density	0.94 (0.82-1.08)	1.04 (0.90-1.20)	0.98 (0.83-1.15)
Spatial Density	0.97 (0.88-1.08)	0.99 (0.89-1.10)	1.03 (0.92-1.16)
**Category E: Health Services**			
Population Density	0.78 (0.64-0.94)	1.00 (0.84-1.19)	0.89 (0.72-1.11)
Spatial Density	0.77 (0.65-0.91)	0.98 (0.83-1.15)	0.93 (0.78-1.12)
**Category F: Food Resources**			
Population Density	0.83 (0.72-0.95)	0.97 (0.84-1.11)	0.97 (0.83-1.14)
Spatial Density	0.88 (0.80-0.97)	0.95 (0.86-1.05)	1.01 (0.91-1.13)
**Category G: Personal Care Services**			
Population Density	0.83 (0.72-0.96)	1.01 (0.87-1.17)	0.96 (0.82-1.14)
Spatial Density	0.85 (0.74-0.97)	1.02 (0.88-1.18)	1.01 (0.86-1.18)
**Category H: Social Infrastructure**			
Population Density	0.95 (0.87-1.04)	1.04 (0.94-1.14)	0.99 (0.89-1.09)
Spatial Density	0.95 (0.88-1.03)	1.03 (0.95-1.12)	1.00 (0.91-1.10)
**Total Assets**			
Population Density	0.90 (0.80-1.02)	1.04 (0.92-1.18)	0.96 (0.83-1.11)
Spatial Density	0.91 (0.81-1.01)	1.03 (0.91-1.15)	1.00 (0.88-1.13)

a. Unadjusted models include random intercept to account for school-level clustering.

© 2024 Szoko N et al. *JAMA Network Open*.

eTable 5. Associations between Community-Level Asset Density and Mental Health: Adjusted Odds Ratios (95% CIs)[a]

Asset Category	Hopelessness	Non-Suicidal Self-Injury	Suicidal Ideation
**Category A: Transportation**			
Population Density	0.77 (0.66-0.90)	1.02 (0.87-1.21)	0.87 (0.72-1.04)
Spatial Density	0.81 (0.68-0.97)	0.97 (0.84-1.12)	0.97 (0.83-1.14)
**Category B: Education**			
Population Density	0.79 (0.67-0.93)	1.00 (0.84-1.18)	0.88 (0.74-1.06)
Spatial Density	0.87 (0.75-1.00)	0.97 (0.85-1.11)	0.99 (0.86-1.14)
**Category C: Parks and Recreation**			
Population Density	0.87 (0.73-1.04)	1.05 (0.89-1.24)	0.93 (0.78-1.12)
Spatial Density	0.90 (0.76-1.07)	1.01 (0.87-1.18)	1.02 (0.87-1.20)
**Category D: Faith-Based**			
Population Density	0.86 (0.72-1.03)	1.10 (0.93-1.29)	0.94 (0.78-1.12)
Spatial Density	0.94 (0.83-1.07)	1.02 (0.91-1.16)	1.03 (0.90-1.17)
**Category E: Health Services**			
Population Density	0.75 (0.60-0.93)	1.03 (0.85-1.25)	0.85 (0.67-1.08)
Spatial Density	0.73 (0.56-0.96)	1.01 (0.84-1.20)	0.94 (0.77-1.15)
**Category F: Food Resources**			
Population Density	0.93 (0.80-1.08)	0.95 (0.82-1.10)	0.98 (0.84-1.14)
Spatial Density	0.89 (0.80-1.00)	0.95 (0.85-1.06)	1.02 (0.90-1.14)
**Category G: Personal Care Services**			
Population Density	0.97 (0.84-1.12)	0.96 (0.83-1.11)	0.99 (0.85-1.16)
Spatial Density	0.89 (0.72-1.10)	1.00 (0.83-1.19)	1.05 (0.87-1.27)
**Category H: Social Infrastructure**			
Population Density	0.93 (0.82-1.06)	1.04 (0.94-1.15)	0.97 (0.87-1.08)
Spatial Density	0.95 (0.87-1.04)	1.04 (0.95-1.13)	0.99 (0.90-1.09)
**Total Assets**			
Population Density	0.86 (0.74-0.99)	1.05 (0.92-1.21)	0.93 (0.80-1.08)
Spatial Density	0.82 (0.65-1.04)	1.04 (0.91-1.18)	0.98 (0.86-1.13)

a. Alternative multilevel models include: fixed effects for asset density, participant age, sex at birth, race/ethnicity, identification as a sexual/gender minority, and COI score (nationally normed); a random intercept to account for school-level clustering; and a random slope for asset density across zip codes. Likelihood ratio tests comparing models with and without a random slope were not significant; thus, these models were not selected for our primary analysis.

© 2024 Szoko N et al. *JAMA Network Open*.

eFigure 3. "Bright Spot" Analysis: Asset Density Versus Proportion of Youth Reporting Hopelessness

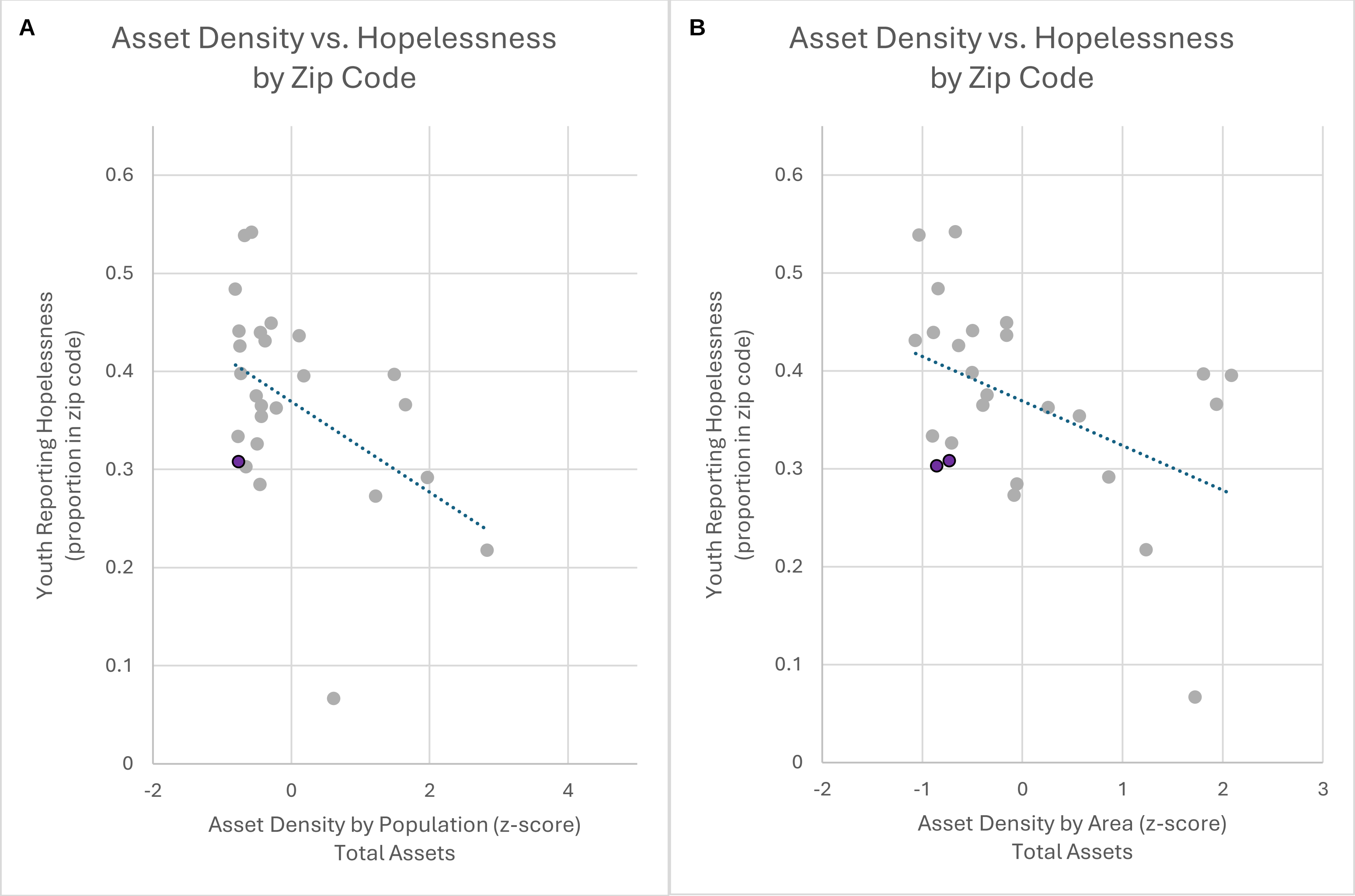

Legend (eFigure 3): Panels A & B plot the population density (A) and spatial density (B) of assets versus the proportion of youth reporting hopelessness across the 26 zip codes examined in the study (trend line (blue)). Two zip codes (15204 and 15214; indicated with purple marker) had both asset density and proportion of youth with hopelessness in the lowest quartile of the sample. The overall COI scores were 11 and 23 (out of 100) for zip codes 15204 and 15214, respectively.

© 2024 Szoko N et al. *JAMA Network Open*.
