# Supplementary material for: Community Asset Density and Past-Year Mental Health Symptoms Among Youths
Source: JAMA Netw Open. 2024 Sep 20;7(9):e2434923. doi: 10.1001/jamanetworkopen.2024.34923 (PMC11415787; doi:10.1001/jamanetworkopen.2024.34923)
Supplement: Supplement 2. — Data Sharing Statement [file jamanetwopen-e2434923-s002.pdf]

Data Sharing Statement

Szoko. Community Asset Density and Past-Year Mental Health Symptoms Among Youths. *JAMA Netw Open*. Published September 20, 2024. doi:10.1001/jamanetworkopen.2024.34923

Data
Data available: No

Additional Information
Explanation for why data not available: A data dictionary for the YRBS survey will be made available upon request from the primary author (nis165@pitt.edu). All other data used in this manuscript are available via publicly accessible online repositories.
